# Antimicrobial Resistance Situation in Indonesia: A Challenge of Multisector and Global Coordination

**DOI:** 10.1155/2022/2783300

**Published:** 2022-02-07

**Authors:** Selma Siahaan, Max J. Herman, Nyoman Fitri

**Affiliations:** ^1^Centre for Research and Development of Humanism and Health Management, National Institute of Health Research and Development, Jakarta 10560, Indonesia; ^2^Centre for Research and Development of Health Resources and Services, National Institute of Health Research and Development, Jakarta, Indonesia; ^3^Centre for Research and Development of Biomedical and Basic Health Technology, National Institute of Health Research and Development, Jakarta, Indonesia

## Abstract

High levels of antimicrobial resistance (AMR) in Indonesia are caused by the use of inappropriate antimicrobials (AM) in healthcare services and the livestock and fisheries sector. The available data and information about overused antibiotics and the AMR threat in Indonesia are limited. The aim of the study is to describe the AMR situation in Indonesia based on perceptions of government officials, health professionals, and the community to determine actions needed to develop AMR-related strategy and policy. The study was done in eight provinces in Indonesia and included reviewing AMR-related policy, collecting antibiotic use reports in primary health care from health offices and hospitals, and conducting in-depth interviews and focus group discussions with informants from health and nonhealth sectors. The results of the study show that AM misuse happens not only in healthcare facilities but also in communities. Medical officers are unfamiliar with AMR-related policy, as are officers in the livestock and fisheries sectors. There is limited coordination between sectors regarding the AMR situation in Indonesia. The government has to take stronger measures to oversee better implementation of AMR policies.

## 1. Introduction

Antimicrobial resistance (AMR) is a global public health concern. The World Health Organization (WHO) reports that AMR constitutes a serious threat to public health worldwide [1]. According to WHO, the world has ran out of effective ways to treat common infections such as urinary tract infections or some form of diarrhea. This situation is indicated by the high levels of antimicrobial resistance to drugs that are often used to treat such infections. The WHO further noted that the level of resistance to ciprofloxacin, an antimicrobial that is often used to treat urinary tract infections, ranged from 8.4% to 92.9% in 33 countries. Recently, improper use of antibiotics during the current pandemic has contributed to making this situation even worse [2].

National data regarding AMR in Indonesia refers to Global Antimicrobial Resistance and Use Surveillance System (GLASS). Indonesia just started joint GLASS in 2019. The data for GLASS collection was limited to 20 selected hospitals spread in Indonesia. These hospitals act as sentinel sites for AMR data. The result from GLASS reported in 2019 for Indonesia was an increase in the percentage of antimicrobial resistances for some bacteria such as *E.coli* and *K. pneumonia*. Those include Carbapenems, Fluoroquinolones, and third generation Cephalosporins [3]. A surveillance study conducted in 2017 on *Escherichia coli* and *Klebsiella pneumoniae*, which caused UTIs, revealed that the resistance to commonly available treatments was quite high in Indonesia [4]. All of this indicated that Indonesia has a quite high level of AMR as other Southeast Asian countries such as India and Bangladesh [1]. This is caused by the use of inappropriate antimicrobials (AM) in the healthcare services and livestock and fisheries sector [5, 6]. In urban areas, people have “easy access” to AM, and it is commonly thought that AM therapy is a cure for several common diseases such as influenza, diarrhea, and others. Moreover, people can purchase antibiotics in drug stores and pharmacies without prescriptions, even though this practice is against government regulations [7–9]. Another contributing factor is noncompliance, which is common in short- and long-term antibiotic therapy for patients suffering from chronic diseases such as tuberculosis and HIV [10]. The practice of using antibiotics irrationally creates favorable conditions for microorganisms to develop resistance and trigger the emergence of antibiotic-resistant microorganisms [11].

The availability of data and information about the role played by misuse of antibiotics in the AMR threat in Indonesia is limited. Basic national health research in Indonesia showed that around 10% of communities kept antibiotics in their homes, and 86.1% obtained these antibiotics without a doctor's prescription [12]. Another study reports that there is a statistically significant increase in the prevalence of infections caused by extended-spectrum beta-lactamases (ESBL) producing bacteria in hospitals; for example, among *Escherichia coli* and *Klebsiella pneumoniae* the average prevalence rate of resistance ranged from 26% to 56% [6].

In relation to the situation outlined above, the Ministry of Health (MOH) issued several policies such as “General Guidelines of Antibiotic Use” (MOH Decree No. 2406/2011). This guideline is a reference for practitioners in health care services for rational use of antibiotics. The latest policies issued are MOH Decree No. 02.02/2014 “Formation of Antimicrobial Resistance Control Committee” and MOH Decree No. 8/2015 “Antimicrobial Resistance Control Programme in Hospitals.” These policies aim to control AMR in health care facilities. Furthermore, in the livestock sector, there is the Ministry of Agriculture Decree No.18/2009 “Animals Medicines and Feed Addictive” that intents to protect consumers from veterinary drugs including antibiotics that do not meet the requirements quality, efficacy, and safety. In the fisheries sector, there are the Ministry of Marine Affairs and Fisheries Decree No.52/2014 “Classification of Fish Medicines” and Decree No.02/2007 “Good Fish Cultivation.” This decree aims to provide assurance of quality assurance and safety of fishery products from the dangers of fish drug residues, chemicals, biological materials, and contaminants, so that fish cultured results are safe for human consumption.

The WHO states that, to overcome the current AMR situation, participation and collaboration are required from all stakeholders; therefore, governments and people have to work together comprehensively [1]. This study aims to describe the AMR situation in Indonesia based on the perceptions of government officials, health professionals, and the community.

## 2. Materials and Methods

### 2.1. Sampling and Data Selection

The study consisted of several activities, i.e., the review of existing antimicrobial resistance-related policy documents, a qualitative study by means of in-depth interviews and focus group discussions with informants from the health and other sectors and hospital patients, and collection of antibiotic use reports in primary health care (PHC) from district health offices and hospitals.

### 2.2. AMR-Related Policy Documents

The review of existing antimicrobial resistance-related policy documents took several steps: firstly, collecting documents, especially documents that are already legalized by the government by online research and in governments offices such as Ministry of Health, Directorate General of Livestock and Animal Health, Ministry of Agriculture, and Ministry of Marine Affairs and Fisheries. Then, learn about these documents including their purposes and goals. After that, highlight documents' contents or issues related to AMR control. Discuss the findings from documents and the study results within the team.

### 2.3. Study Sites and Sample Locations

The study sites and sample locations were selected purposively. The study sites were located in eight provinces in Indonesia, which were categorized into four parts: DKI Jakarta represents the capital city; DI Yogyakarta, East Java, and North Sumatra represent the western part; Bali and South Kalimantan represent the central part; South Sulawesi and East Nusa Tenggara represent the eastern part. In each province, we collected data from urban area, i.e., capital city of the province, and the rural area; thus, there were 16 districts in the eight provinces.

The sample locations selected met the following criteria: one (1) public hospital in urban, one (1) private hospital in urban, and one (1) public hospital in rural areas, because not all of the rural areas have private hospitals. Other sample locations were two (2) primary health cares that are located around public hospitals. Furthermore, sample locations also involved the provincial and district health offices, fisheries offices, and livestock offices.

This study was conducted between September 2014 and June 2015. However, the authors in writing this manuscript also considered the current situation of AMR in Indonesia.

The interview with informants in the health, livestock, and fisheries sectors lasted between one and two hours, depending on the informant's level of knowledge, understanding, and interest. The FGDs lasted approximately two hours. The interview and the FGDs were structured around the following themes: “AMR situation and current use of AM”; “the policy related to AMR and its technical policy and guidelines”; “the communication of the policy and its target”; “implementation of the policy and its problems”; and “monitoring and evaluation.” An in-depth interview was also conducted to the patients/patients' family members in the hospital.

A written consent agreement was requested from the informants before the interviews began in accordance with the direction of the ethics committee of the National Institution of Health Research and Development, which issued the ethical clearance for this study.

### 2.4. Qualitative Study

This qualitative study aimed to explore the AMR situation and its related problems in Indonesia and to determine what actions are needed in order to develop strategy and policy related to AMR. Data and information were collected through focus group discussions (FGD) and in-depth interviews as follows.

### 2.5. Focus Group Discussion with Health Practitioners

FGD was conducted with two types of groups. Group I consisted of participants who were responsible for the AMR program in hospitals. In this group, the participants were medical doctors and pharmacists, as they are health professionals who were appointed by their hospital directors to be responsible for AMR control programs in hospitals. The FGDs for this type of group were conducted in the provincial health offices. Six persons, each representing a different hospital, participated in the FGD in each province. The total number of participants in these focus groups in the eight provinces was thus 48. Group II consisted of medical doctors who work in primary health care facilities. Between six and eight persons from six and eight primary health care facilities participate in the FGD in each province. The total number of participants in this type of group in the eight provinces was 56.

### 2.6. In-Depth Interviews with Programmes Managers in Health Sectors

In-depth interviews in the health sector: the informants were two program managers who were responsible for the rational drug use program in the Directorate General of Pharmaceutical Affairs and Drug Control, Ministry of Health, and 24 program managers from provincial and two district health offices (urban and rural).

### 2.7. In-Depth Interviews with Programmes Managers in Livestock Sectors

In-depth interviews in the livestock sector: the informants were two program managers who were responsible for the healthy meat consumption program in the Directorate General of Livestock and Animal Health, Ministry of Agriculture, and 23 program managers from provincial and district livestock offices. We also included three persons from research centers that conduct research into local meats. These research centers, which operate under the Ministry of Agriculture, were located at the study sites. There were research centers in only two of the provinces visited; the other six provinces did not have such research centers. The purpose of these centers is to examine the local meats for research.

### 2.8. In-Depth Interviews with Programmes Managers in Fisheries Sectors

In-depth interviews in the fisheries sector: the informants were two program managers who were responsible for the healthy fish consumption program in the Directorate General of Fisheries Production, Marketing and Processing, Ministry of Marine Affairs and Fisheries, and 22 program managers from provincial and district fish offices. Likewise, we included four persons from three research centers that conduct research into local fishes. These research centers, which operate under the Ministry of Marine Affairs and Fisheries, were located at the study sites. There were research centers in only three provinces visited, four provinces did not have research centers, and one research center was not available to be interviewed. These centers examine the local fishes for research.

### 2.9. In-Depth Interviews with Patients

In-depth interviews with patients (or patients' families) were held in three hospitals in each province. The number of patients was 10 to 12 persons for each hospital. The total number of patients interviewed was 271 persons.

Patients/patients' family members were selected for interviewing based on the criteria that they were outpatients who had recently received antibiotics from the hospital pharmacy and were voluntarily available to be interviewed. The patients' ages ranged from 17 to 65. Each interview lasted between 20 and 30 minutes. Age 17 in Indonesia is an adult age. At the age of 17, a person will get an identity card from the government. The interviews were structured around topics such as “the experience of patients using antibiotics”; “knowledge of patients about antibiotics, such as how to consume antibiotics”; “source of information of antibiotic knowledge”; “antibiotics are used for what kind of diseases”; “what are the dangers of consuming antibiotics”; “how can one obtain antibiotics, can one purchase it without a doctor's prescription”; and “the practice of consuming antibiotics.”

### 2.10. Data Analyses

Researchers transcribed the information collected, then summarized it. A theme-based approach (thematic analysis) was applied. Key information was entered into a matrix and coded; highlighting with several colors was used to identify different themes, opinions, and language. Researchers did it manually. This matrix was discussed within the study team several times. Themes resulting from these analyses can be seen in appendix C. Triangulation was applied using multiple sources, such as document reports, documents policies, and compatibility between information from different informants in order to improve the objectivity and internal validity. After that, it was discussed with academics who are experts in public health policy, which resulted in several optional conclusions and recommendations. Finally, a round table discussion was organized with stakeholders from government (central and local), universities, health providers, and community organizations to determine the best possible policy recommendation for AMR situation in Indonesia.

## 3. Results and Discussion

The results of the study that will be shown based on in-depth interview with program managers in health, livestock, and fisheries sectors. Those also include the results of FGDs with health practitioners in hospitals and primary health cares. The results of patients' interview will also be shown. Other than qualitative information, the pattern of AM uses in hospital and primary health care for three (3) years period will be displayed.

### 3.1. Results

In-Depth Interviews and Focus Group Discussions

#### 3.1.1. Health Sector

AMR situation and current use of AM: most informants in health offices, hospitals, and primary health care facilities confessed that controlling of AM use had not worked properly either in health care settings or in communities. One thing that causes this is “easy access” to AM due to weak oversight, with the result that people can buy antibiotics without prescriptions and even purchase them in drugstores, which is prohibited by regulations.

Within the community, knowledge of, attitudes toward, and practices regarding AM use were limited and incorrect. In an FGD forum in April 2015, for example, a doctor from a primary health care facility in Bogor, West Java, said:

“Sometimes patients get angry at us because we did not prescribe antibiotics for their therapy, they think that antibiotics are the best medicines for their diseases.”

The policies related to AMR and its socialization: to control AMR-related problems, the government has issued several policies and guidelines, as mentioned above. However, many doctors and pharmacists who work in district health offices, hospitals, and PHCs are unaware of these policies. They are only familiar with the National Formulary, as all prescribed medicines are associated directly with a payment mechanism system for National Health Insurance. It can be said that the policies, including the guidelines, have not been communicated well.

Implementation of the policy and its problems: only a few large public hospitals already have a program of antimicrobial controls, as it is mandatory for hospital accreditation assessment and also regulated in MOH Decree No. 8/2015 “Antimicrobial Resistance Control Program in Hospitals,” while many other hospitals do not have such a program. Doctors and pharmacists in hospitals said that there was a lack of support from the directors and management of hospitals to implement the antimicrobial program just like in hospital in Kenya [13]. Doctors are still allowed to use high-line antibiotics such as meropenem without a preceding antibiotic sensitivity test. The reagents for the sensitivity tests are considered expensive, so that there are sometimes no reagents for this test. In addition, there are no reward and punishment schemes for rational drug use in hospitals.

The health professionals in primary health care blamed the drug supply management from district health offices since medicines for PHCs are mostly distributed from local health offices. The availability and the efficacy of medicines were lacking. The PHCs created the medicine plans that were suited to their needs and submitted them to the district health offices. Then, health offices procured the medicines and delivered them to the PHCs. However, based on FGDs, many of the medicines, including antimicrobials, were given only in limited amount or not enough to fulfil the need of the PHCs and the patients. Accordingly, for example, amoxicillin, which should be prescribed for five days to the patients, were given for only three days. A pharmacist from a primary health care in Bali district told the researchers in FGD forum in March 2015 what follows:

“… we tried to make a good medicine plan for our needs, however it did not surprise us when only small quantities of medicines (including antibiotics) came from the district health office.”

Doctors and pharmacists in PHCs and hospitals need continuing education for better implementation of the rational use of medicines, including antibiotics, especially for freshly graduated doctors [11]. There are training courses on the rational use of medicine programs of the MoH; however, this program only reaches pharmacists since only pharmacists are trained. In this program, pharmacists are obliged to make routine reports about the rational use of antibiotics for acute nonpneumonia respiratory diseases and nonspecific diarrhea. An evaluation of this program, conducted by National Institute of Health Research and Development (NIHRD), showed that the achievement of the rational drug use indicator was considerably lower than its target [14]. It would be better if the MoH presented the training on rational use of medicines not only for pharmacists, but also for doctors in PHCs. Lack of pharmacists in PHCs was also complained about, because it often happened that drug management and drug services were performed by persons without a pharmacy background. Many of them graduated from senior high school and were being informally trained for drug services. If drugs are delivered to patients by a person without a pharmacy background, then there will be no quality control processes such as appropriate drug storage, drug dosages, and drug interactions [15].

Monitoring and evaluation: monitoring of Guideline about Rational Use of Medicines including antibiotics for primary health cares was conducted routinely by district health offices. Evaluation refers to the subject about the compliance of practitioners to prescribe medicines that have to refer to national formulary. However, most of health care services visited by researchers had not conducted evaluation about AM use, except the hospitals that have already implemented AMR control program.

The pattern of AM use in hospitals and primary health care facilities from 2012 to 2014 is shown in Tables 1 and 2. From these tables, we can see that, only in a three-year period, the use of antibiotics in health care services already showed a tendency of an increase in the use of higher-line generation. In 2014, cefixime became the first most used antibiotic in several hospitals, and meropenem was included in the ten most used AM by hospitals in 2013 and 2014. Ciprofloxacin and clindamycin were included in the ten most used antibiotics in PHCs.

#### 3.1.2. Livestock Sector

As in the health sector, the distribution of AM for animals was poorly controlled. According to regulations, the use of AM for animals can be prescribed only by veterinaries; however, this guideline is not implemented well. Many farmers give AM to their livestock by themselves, because it is easy to obtain AM and it seems that no one cares about these practices. In addition, the large number of small farms makes it difficult to control them.

There are also feed additives containing growth promoters for chickens. These products actually go against regulations. In addition, in several places, penicillin and streptomycin resistance has been found in pigs. Veterinary research centers also report that there are many animal products that contain antibiotic residues. However, problems related to AM residues in the livestock sector have not been prioritized, as this sector is still focusing on other safety issues, such as misuse of formalin, borax, and hormones in animal products as the following quotation from a program manager in Ministry of Agriculture makes clear:

“… our program focused on meats for domestic consumption … related to the healthy meats, we tried to minimize formalin and borax used in meats, we have not reached to antibiotics residue control …”

Several policies and guidelines have been issued by the government in order to control AMR in animals, e.g., Law No. 41/2014 about livestock and animal health, Law No. 18/2009 about animal's medicines and feed additives, and other policies relating to general meat safety and health, and halal meats. There are several underlying causes for the policies not being effective. Communication of the policies does not go smoothly because of the decentralization of the tiered regions. From the center to the provinces, the communication is quite good, but from province to district it is still questionable because in Indonesia decentralization is on the district levels. It creates a situation in which each district tends not to follow central policies in terms of controlling their own community. The number of veterinarians who work in government sectors is also still limited, and these veterinarians are needed for providing coaching and supervision to farmers. The implementation of policies regarding food safety sometimes contradicts policies about food security and economic development; for example, national need for meats is the first priority; then, healthy meats will be the next priority.

#### 3.1.3. Fisheries Sector

The situation in the fisheries sector is a bit better when compared to the other two sectors mentioned before, especially regarding fishes and shrimps intended for export. This is because the inspection of fishes and shrimps for export is rigorous. The quarantine centers conduct monitoring and evaluation every six months. Of special concern are fishes and shrimps for domestic consumption since they are not under as tight control as export products. An informant from the district fisheries office in South Sulawesi said:

“ … we did not inspect fisheries for local market as tightly as for export.”

There are inspections for AM residues in shrimps in shrimp farms every year. These activities can only be done by quarantine centers and universities since district fisheries offices have no capability to do the inspection of AM residues due to lack of facilities, skilled persons, and other resources. Several informants said that, based on their annual reports, the most significant residue of AM found in fishes was chloramphenicol. Once again, easy access to AM and the large number of small fish farms are the principal underlying factors for residual AM in fishes.

One of the policies related to AMR in the fisheries sector is Law No.45/“Fisheries.” It states that the use of several medicines is prohibited, for example, chloramphenicol and nitrofuran. Another policy is Ministry of Marine Affairs and Fisheries Decree No.02/2007 “Good Fish Cultivation.” The informants stated that communication of the polices was conducted directly to the stakeholders, at least once a year. The contents of the communications were about the regulations, fish medicines, and chemical and biological substances that are allowed or prohibited in the fisheries sector. However, there were still farmers, especially small farmers, who have not understood the policy well.

#### 3.1.4. Patient/Patients' Family Members

The number of patients or patients' family members interviewed was 271. The age of these informants interviewed ranged from 17 to 65. The education of these informants was high school (152 persons) and university (89 persons), and the rest only had an elementary educational background. Based on the analyses of the in-depth interview, results lead to three (3) main themes. Theme 1 is “Knowledge of patients about AMR and AM use”; this can be seen from the following information. There were 145 who said that they knew that a course of antibiotics had to be finished. Only 65 persons said that antibiotics could be used for people with infectious diseases, and 62 persons said that there was a risk of resistance if antibiotics are not used properly. There were also 65 persons who stated that antibiotics could only be purchased based on a doctor's prescription.

Theme 2 is “Compliance to the dosage of antibiotics as doctor's prescription.” This leads to the following information. There were 151 persons who said that they complied with it, while the rest of them only took the antibiotic until they feel healthier. Theme 3 is “The source of information about antibiotics.” Many of the patients got information of antibiotics from health professionals such as doctors, pharmacies, and nurses (191 patients), while others reported getting information from media, family, friends, and educational institutions.

### 3.2. Discussion

The study result shows that the irrational use of AM occurred not only in health care facilities (primary health care centers and hospitals), but also in communities. In the livestock and fishery sectors, the irrational use of AM occurred mostly on the farms. This is similar to the situation in other developing countries such as India, Philippines, and Vietnam [16, 17].

In the pandemic era, the use of antibiotics is more uncontrolled. Digitalization and telemedicine are health care services that have suddenly emerged and are widely used by the public; on the other hand, private providers use this opportunity to gain profits. Many people infected with COVID-19 chose to self-isolate, and they received telemedicine treatment, and some of them receive azithromycin treatment if the symptoms of Covid-19 are accompanied by fever. Telemedicine in Indonesia is popular based on applications and makes it much easier for patients to get access to treatment and medicines such as azithromycin. Meanwhile, the government has not issued regulations related to application-based telemedicine treatment, so that the purchase of antibiotic drugs can occur without the supervision of qualified health workers and the government [18].

Problems related to irrational use of AM in health care facilities are due to several factors. The policies and guidelines related to the use of AM have not been communicated well. Many doctors and pharmacists did not know about these policies. Kakkar et al. (2018) stated that several countries in South East Asia have some policy systems to overcome AMR; however, this is not supported by mature implementation plans and monitoring systems [19]. The availability of antibiotics such as amoxicillin in primary health care facilities was inadequate [13]. Drug management, supply, and distribution in district health offices are not run well. It can be said that inappropriate medicine plans and management lead to irrational use of AM [15, 20]. The incompetence of health workers such as doctors and pharmacists is another important factor. They need to be trained continuously [11]. The training and socialization about AMR and its policy can be easier and cheaper and reach more health workers now, as long-distance learning (online) becomes popular and acceptable in the COVID-19 situation.

The study also showed that amoxicillin was the AM that was most used in health facilities and communities. However, there was high resistance to amoxicillin [21]. Study results also showed that, only in a three-year period, the use of AM in health care services tended to change to the higher-line antibiotics such as ciprofloxacin and clindamycin, which were included in the ten most used AM in PHCs. These AM are not used for basic health care services based on primary health care standard therapy. Inappropriate use of antibiotics occurs in many low and middle income countries [22].

Study meropenem was included in the ten most used AM in hospitals. Many of the cases in which meropenem was used were not based on preceding sensitivity test results. In other words, meropenem was used for empirical therapy. A study at Sultan Qaboos University Hospital (SQUH), Muscat, Oman, showed that meropenem was prescribed empirically in 382/400 (96%) of the cases [23]. Based on this situation, it can be said that the use of the higher-line of AM is due to the occurrence of resistance on the older generation of AM. A study on antibiotics in West Java-Indonesia reported that most of the antibiotics consumed by our population appear to be ineffective although pathogenic bacteria can be found in 21% of cases of acute diarrhea cases in the hospitals due to high resistance rates of enterotoxigenic *Escherichia coli* (ETEC) [24]. This trend may change due to COVID-19 situation. Until mid-2021, In Indonesia, most of health care facilities focus more on COVID-19 therapy, especially public facilities. In the beginning of the pandemic era, the COVID-19 therapy involved hydroxychloroquine as a drug of choice following WHO guidelines at that time. However, later on, hydroxychloroquine was no longer allowed to be used for COVID-19 treatment because of its ineffectiveness and safety reasons [25]. The inappropriate use of AM including azithromycin and hydroxychloroquine may lead to the worse AMR situation in Indonesia.

Problems related to the irrational use of AM in communities, farms, and the livestock and fisheries sectors are in consequence of several factors. Easy access to AM occurred because of poor oversight. A survey conducted by Purwaningsih on 16 chicken farmers in Jakarta regarding the use of antibiotics in chickens showed that 31.30% of farmers admitted to using antibiotics for their livestock with the reasons that the chickens would be healthy and fat, while the rest of them were because the chicken is sick. Similarly, adherence to antibiotics based on a veterinarian's prescription was only 25%, while others said that antibiotics were only given when their livestock showed signs of sickness [26].

The regulations and policies of AMR in the central government have not been synchronized with regulations in the district level. The regulations related to AMR control should be adopted and integrated in regional regulations; thus, this will be more straightforward [19]. According to the informants, the problems of oversight in farms are because there are so many small farms spread throughout their provinces. In addition, they also stated that the number of field inspectors for controlling the farms was inadequate. The government should find a way to solve this situation. For example, responsible farmers can be appointed to assist the government. Several farms can be gathered into one group that is supervised by an appointed farmer. Related to this, improving knowledge, attitudes, and practices of farmers about antibiotics is indispensable; thus, a supportive environment is needed. This could include regulations to control use, better systems for monitoring use, and financial incentives for the farmers [27, 28]. In addition to the fisheries sector, the government should also pay attention to fishes and shrimps for domestic needs. The solid control and inspection should also be implemented as for fishes farmed for exporting [29].

Community perception as well as their knowledge, attitudes, and behavior in terms of AM use is still incorrect. This similar situation also happened in Poland and Lithuania [30, 31]. This is very common in developing countries, even in an industrial country like China [21, 32, 33]. It would be better if the knowledge related to AMR was introduced at an early age, for example, as a topic for a school lesson at the high school level. In Indonesia, drugstores are not allowed to sell antibiotic according to government regulations. However, many people can buy antibiotics in drugstores. Enforcing the law for antibiotic distribution should be carried out. If not, it is extremely difficult to control AMR [34].

An information system for AMR has not been established. If there was an interconnected information system, it could warn health workers to be more aware about the AMR problems and could lead to responsible behavior regarding using or prescribing AM [17].

There was little (no) coordination between sectors in relation with the AMR situation in Indonesia [35–37]. There are also no AMR indicators bound to the programs that are useful to assess the level of AMR from time to time [38].

**Figure 1:**
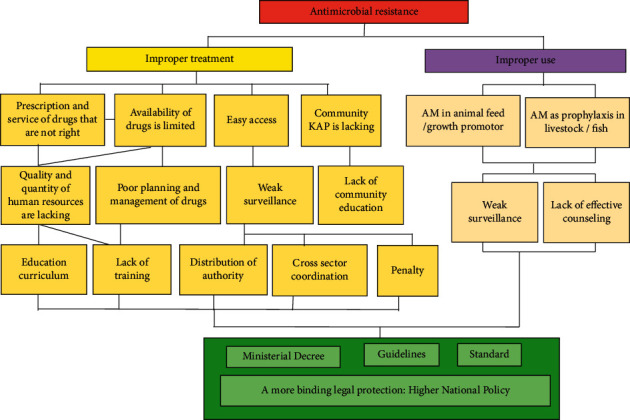
Tree analysis of AMR situation in Indonesia [39]. ^*∗*^KAP: Knowledge, Attitude and Practice.

Furthermore, the results of analyzing the AMR situation using tree analyses are shown in Figure 1. Tree analysis is a method used to find a solution to a problem by identifying and mapping out the anatomy of cause and effect around the problem. The question “Why?” is asked many times throughout the process, and the root cause and ultimate effects can be presented in the form of a tree (Figure 1) [39].

It was clear from the tree analyses that since AMR problems bond to multiple sectors, the solution to these problems has to be multisectorial, too. In China, the leading cause of AM resistance is the irrational use of antimicrobial agents in healthcare and veterinary environments as well as the use by the general public. The same study also reveals that, in the last ten years, the Chinese health administrative authorities have been unsuccessful in coping with this problem due to lack of mandatory regulations [33]. Nowadays, Indonesia is facing similar situation as China.

In the COVID-19 era, the AMR situation is getting worse. At present, many experts are concerned that global efforts to control AMR may encounter setbacks during the COVID-19 pandemic. This can be seen from the existence of premature trends regarding the benefit of using antibiotics for COVID-19 therapy, such as taking azithromycin in combination with hydroxychloroquine. Moreover, shortage will occur as a result of a massive use of the drugs [40].

The policy about AMR control is still stagnant in Indonesia. The National Committee of Antimicrobial Resistance Control, a committee under the Ministry of Health, is still unable to tackle AMR. The sectors that are accountable to this committee work by themselves to deal with AMR. Coordination with other sectors might appear easy on paper, yet hard to do. Indonesia still needs a robust legal framework that can bind multisector to tackle AMR simultaneously. The harmonization between central and regional/district governments also needs to be strengthened. Likewise, the AMR policy interventions in both governments need to be compatible. Therefore, Indonesia needs an approach in health settings to integrate all related sectors. This approach can be in the form of national policies that cover multiple sectors and can be performed at the regional level. Collaboration with other countries is also needed as an important support to implement “One Health” [11].

### 3.3. Limitation of the Study

The study has several limitations: the information collected depended on the knowledge and interest of the informants, and the supporting documents (especially formal reports that consist of surveillance data and local policies) were not always available.

## 4. Conclusions

The government faces a big challenge in overcoming the AMR problem in Indonesia. The AMR situation in Indonesia is not getting any better; in fact, it has worsened. Policies related to AMR are limited, and those alone cannot solve the AMR problem. The communication of these policies has not reached its targets. Many doctors, pharmacists, and even district health officers are unaware of these policies. Accordingly, the implementation of these policies cannot work properly.

The study results for the livestock and fisheries sectors also showed a similar situation. The policies relate to AMR were known partially by their targets, such as officers who work in district offices.

There was little (no) coordination between sectors in relation to the AMR situation in Indonesia. In relation to easy access due to weak oversight, the government has to take stronger measures to oversee better implementation of AMR policies.

### 4.1. Recommendation

Further research, for example, on the economic impact of AMR, will complement this study.

The government should issue a national policy that directly addresses the solution of the AMR situation in Indonesia. This policy should be an umbrella for all related sectors and can be integrated into regional policies. It also needs global collaboration to support the implementation of the policies.

## Figures and Tables

**Table 1:** The ranking of 10 antimicrobials mostly commonly used in hospitals (16 districts of 8 provinces).

No.	Year		
	2014 (*n* = 19)	2013 (*n* = 24)	2012 (*n* = 22)
1	Cefixime	Amoxicillin	Amoxicillin
2	Amoxicillin	Cefadroxil	Cefadroxil
3	Ceftriaxone	Ciprofloxacin	Ciprofloxacin
4	Ciprofloxacin	Metronidazole	Ceftriaxone
5	Metronidazole	Ceftriaxone	Metronidazole
6	Clindamycin	Cefixime	Cotrimoxazole
7	Cotrimoxazole	Cefotaxime	Erythromycin
8	Doxycycline	Clindamycin	Doxycycline
9	Cefotaxime	**Meropenem**	Cefixime
10	**Meropenem**	Ethambutol	Cefotaxime

*Note. n* = number of hospitals where data were collected and compiled.

**Table 2:** The ranking of 10 antimicrobials mostly commonly used in primary health cares (15 districts of 8 provinces).

No.	Year		
	2014 (*n* = 15)	2013 (*n* = 15)	2012 (*n* = 15)
1	Amoxicillin	Amoxicillin	Amoxicillin
2	Cotrimoxazole	Cotrimoxazole	Cotrimoxazole
3	Ciprofloxacin	Ciprofloxacin	Ciprofloxacin
4	Clindamycin	Erythromycin	Erythromycin
5	Erythromycin	Acyclovir	Acyclovir
6	Lincomycin	Metronidazole	Tetracycline
7	Tetracycline	Tetracycline	Chloramphenicol
8	Chloramphenicol	Griseofulvin	Ampicillin
9	Albendazole	Chloramphenicol	Metronidazole
10	Ampicillin	Doxycycline	Thiamphenicol

*Note. n* = number of district health offices such as PHC where data were collected and compiled.

## Data Availability

The data used to support the findings of this study may be released upon application to the Head of Centre for Research and Development of Humanism and Health Management, National Institute of Health Research and Development Republic of Indonesia, who can be contacted at pusathumaniora@yahoo.co.id.

## Appendix

### A. Interview Guidelines with District Health Office, District Livestock Office, and Districts Fisheries Offices

Informant: program managers related to AMR and AM use in health, livestock, and fisheries sectors.

  Name:
  Institution:
  Work unit:
  Job position (respondent has to be >6 months in this position):
  Age:
  Educational background:
  Phone no.:
  Date & Time of interview:
  Start at:
  Finish at:
  Interviewer

Issues need to be probed.

1. The current situation of antimicrobial and AM use. (Ask informants to explain and provide examples.)
2. Existing policies related to AM use and AMR including their technical instructions. (Policies can be in the form of instructions from the head of the offices, circular letters to follow certain policies, technical guidelines, etc.) (Ask the documents to be showed and take the pictures of them).
3. Socialization of the policy to the targets of the policy. (For the health sector, socialization to the health care services. For the livestock sector to farms and veterinarians. For the fishery sector to fish farms or fishermen. As well as other related units deemed necessary)
4. The implementation of the policy mention before and problems of policy implementation. (Persons/units are involved in implementation, commitment to the implementation, problems and efforts to overcome problems, etc.)
5. Monitoring and evaluation of the policy. (How to do it and the follow-up of the monitoring and evaluation)
6. Suggestion for a better AMR situation in Indonesia

### B. Guidelines for Patients Interview

Informant: patients or patients' family of hospital who have completed treatment, at least 17 years of age and have experience using antibiotics.

  Name:
  Sex:
  Sample location:
  Age:
  Education:
  Job:
  Phone no.:
  Date & Time of interview:
  Start at:
  Finish at:
  Interviewer:

Issues need to be probed.

1. Patients experiences of using antibiotic
2. He/her own experience
3. Family/relative experience
4. Informants' habits of buying medicine (such as buy all medicines, buy only half, where to buy, etc.)
5. Patient compliance to take medication, especially antibiotic drugs (regularly and until the drug runs out)
